# Beyond Thrombus Level: Increased Surgical Complexity, Nutritional Status, and Postoperative Complications Shape Outcomes in Renal Cell Carcinoma with Venous Tumor Thrombus—A 14-Year Single-Center Experience

**DOI:** 10.3390/cancers18172789

**Published:** 2026-08-27

**Authors:** Zuzanna Korbecka, Beata Jabłońska, Michał Serafin, Wiktoria Kuczmik, Bartosz Pomianowski, Karolina Blady, Robert Król

**Affiliations:** 1 Department of General, Vascular and Transplant Surgery, Medical University of Silesia, 20-24 Francuska Street, 40-027 Katowice, Poland; wiktoria.kuczmik@365.sum.edu.pl (W.K.); rkrol@sum.edu.pl (R.K.); 2 Department of Digestive Tract Surgery, Medical University of Silesia, 14 Medyków Street, 40-752 Katowice, Poland; 3 Department of General Surgery, Vascular Surgery, Angiology and Phlebology, Faculty of Medical Sciences in Katowice, Medical University of Silesia, 45-47 Ziołowa Street, 40-635 Katowice, Poland; michal.serafin@interia.com; 4 Department of Human Anatomy, Medical University of Silesia, 18 Medyków Street, 40-752 Katowice, Poland; s83086@365.sum.edu.pl (B.P.); s86570@365.sum.edu.pl (K.B.)

**Keywords:** renal cell carcinoma, renal vein, inferior vena cava, venous tumor thrombus, surgery, nephrectomy, complications, survival, predictive factors

## Abstract

**Background:** Renal cell carcinoma (RCC) with venous tumor thrombus (VTT) (RCC-VTT) represents a surgically challenging entity with heterogeneous prognosis. While the level of thrombus has traditionally been considered a key prognostic factor, its independent impact remains controversial. **Aim:** The aim of the study was to evaluate perioperative and oncological outcomes in patients with RCC-VTT, as well as to assess the impact of VTT level on the surgical and oncological outcomes, and identify predictors of postoperative complications and overall survival (OS). **Material and Methods:** A retrospective analysis of patients who underwent radical nephrectomy with thrombectomy for RCC-VTT between 2012 and 2025 was performed. Patients were stratified into two groups based on thrombus level: low-level (VTT 1) vs. high-level (VTT 2–4) according to Neves–Zincke classification. Clinical, laboratory, and pathological variables were analyzed. Predictive factors for postoperative complications and OS were identified using logistic regression analysis and Cox regression analysis. **Results:** A total of 235 patients were included, with 123 in the low-level group and 112 in the high-level group. Patients with higher VTT level had significantly higher NRS 2002 score (3 vs. 4, *p* < 0.001) and lower preoperative hemoglobin levels (12.21 vs. 11.15 g/dL, *p* < 0.001). High-level VTT was associated with longer operative time (median: 107.5 vs. 125 min, *p* = 0.06), increased blood loss (264.11 vs. 599.18 mL, *p* = 0.001), increased red blood cells transfusion (0.69 vs. 1.46 units, *p* < 0.001) and higher rate of major complications (Clavien–Dindo ≥III: 19 (33.33% vs. 12.50%, *p* < 0.001)). In multivariate analysis, VTT level was not an independent predictor both for postoperative complications. In the multivariate model, the NRS 2002 (OR = 4.90, 95% CI = 2.53–9.48, *p* < 0.001) and blood loss (OR = 1.002, 95% CI = 1.001–1.003; *p* < 0.001) proved to be an independent predictive factors for postoperative complications. In multivariate Cox proportional hazard regression analysis, including only preoperative parameters, the NRS 2002 (HR = 1.44, 95% CI = 1.04–1.99, *p* = 0.03) and N1-2 (HR = 1.67, 95% CI = 1.07–2.61, *p* = 0.02) were independent predictive factors of OS. **Conclusions:** Higher VTT level was associated with increased surgical complexity, including longer operative time, greater blood loss, and higher transfusion requirements, but was not an independent predictor of OS. VTT level was not an independent predictive factor for OS. Long-term survival was independently associated with nutritional status (NRS 2002), and lymph node involvement.

## Simple Summary

Renal cell carcinoma (RCC) with venous tumor thrombus (VTT) (RCC-VTT) represents a surgically challenging entity with heterogeneous prognosis. In this study, we analyzed 235 patients who underwent surgery for RCC-VTT to determine the association between the extent of tumor thrombus and surgical complications and long-term survival. Patients with higher-level tumor thrombus had greater blood loss, required more blood transfusions, and experienced more major postoperative complications. However, the level of tumor thrombus itself was not an independent predictor of postoperative complications or overall survival (OS). Instead, nutritional status, lymph node involvement, and other patient- and treatment-related factors were more important for long-term outcomes. These findings suggest that careful preoperative assessment and optimization of patients, together with effective perioperative management, may be particularly important in reducing complications and improving outcomes in patients undergoing surgery for renal cell carcinoma with venous tumor thrombus.

## 1. Introduction

Renal cell carcinoma (RCC) with venous tumor thrombus (VTT) (RCC-VTT) is a distinct and clinically challenging disease, affecting approximately 4–10% of patients at diagnosis [1,2]. Radical nephrectomy and thrombectomy remains the standard treatment offering the only potentially curative option [3]. However, these procedures are related to substantial perioperative morbidity, reflecting both the technical complexity of surgery and the compromised clinical status of affected patients [4,5].

The extent of venous involvement, typically classified according to thrombus level, has historically been considered a key determinant of both surgical risk and oncological outcomes [6]. According to the literature, higher thrombus levels are generally associated with increased operative complexity, greater blood loss, and worse survival [6,7]. Nevertheless, emerging evidence suggests that the prognostic value of thrombus level may be limited when considered in isolation, as patient-related factors and tumor biology appear to play a critical role in determining outcomes [8].

Recently, increasing attention has been directed toward host-related characteristics, particularly nutritional status and systemic inflammation, as important modulators of surgical and oncological results [9]. While inflammatory markers such as the neutrophil-to-lymphocyte ratio (NLR) have been widely studied, their predictive performance remains inconsistent [10]. In contrast, nutritional risk score 2002 (NRS-2002) may provide a more comprehensive assessment of patient frailty [11]. Despite this, their role in patients with RCC and VTT has not been clearly established.

Furthermore, perioperative factors, including intraoperative blood loss and postoperative complications, are recognized as critical determinants of long-term outcomes in oncological surgery [12]. However, in the context of RCC-VTT, their relative importance remains insufficiently explored, with most studies continuing to emphasize tumor-related characteristics [13].

Given the ongoing controversy surrounding the prognostic impact of VTT level and the evolving therapeutic landscape, a comprehensive evaluation of the association between thrombus extent and clinical outcomes is warranted. Therefore, the aim of the present study was to evaluate perioperative and oncological outcomes in RCC-VTT patients, with a particular focus on the impact of nutritional status and perioperative variables. Specifically, we sought to identify independent predictors of postoperative complications and to assess the effect of postoperative morbidity on overall survival (OS). By doing so, we aim to provide a more integrated perspective on risk stratification that extends beyond traditional anatomy-based models.

## 2. Material and Methods

### 2.1. Patients

Clinical data were collected from 235 adult patients with RCC-VTT who underwent open approach surgical treatment in the Department of General, Vascular and Transplant Surgery, Medical University of Silesia, Katowice, Poland between 2012 and 2025. The study population consisted of 152 (64.68%) men and 83 (35.32%) women, with a median age of 67 IQR 14, (range 30–87) years.

Patients were included if they had locally advanced primary RCC with renal vein (RV) or inferior vena cava (IVC) tumor thrombus and were older than 18 years. There were 10 (4.26%) patients classified as M1 according to Tumor–Node–Metastasis (TNM) classification in the analyzed group. Exclusion criteria comprised RCC without venous tumor thrombus, recurrent disease, and incomplete demographic and/or clinical data.

### 2.2. Study Design

All procedures involving human participants were conducted in accordance with the principles of the 1964 Helsinki Declaration and its later amendments, or equivalent ethical standards.

A retrospective review of medical records was performed. The collected and analyzed data included clinical and pathological parameters such as age, gender, Charlson Comorbidity Index (CCI), body mass index (BMI), nutritional risk score 2002 (NRS 2002) [11], intraoperative blood loss and transfusion requirements, length of hospital stay, duration of surgery, preoperative (0) and postoperative (1) laboratory results, tumor laterality, tumor size, level of VTT, clinical and histopathological stage and grade, postoperative complications, and overall survival (OS).

The level of VTT was classified according to the Neves–Zincke system [9]: level 1—renal vein; level 2—infrahepatic IVC; level 3—retrohepatic IVC; and level 4—right atrium [9]. RCC staging was determined according to the 8th edition of the American Joint Committee on Cancer (AJCC) TNM classification system [10], based on preoperative abdominal computed tomography (CT) findings and postoperative pathological examination. Postoperative complications were reported and divided according to the Clavien–Dindo classification. Histological subtypes were defined in accordance with the World Health Organization classification of tumors of the urinary system and male genital organs (2004 edition) [11,12]. Tumor grade was assessed using Fuhrman’s grading system, the most widely used pathological grading method for RCC, based on nuclear and nucleolar morphology [11,13].

Information regarding OS was obtained from a statistical database.

The analyzed patient group was divided into 2 subgroups according to the VTT level, level 1 (group 1) and levels 2–4 (group 2), and multiple clinico-pathological features were compared between these two VTT level groups.

### 2.3. Surgical Technique

The surgical procedure was performed as follows: A laparotomy through a transverse subcostal incision was carried out. The right/left renal artery was dissected and transected, followed by dissection and transection of the right/left ureter. In patients with adrenal metastases or direct tumor invasion, an ipsilateral adrenalectomy was performed.

For Neves–Zincke level 1 cases, the IVC was exposed and partially clamped using Satinsky forceps. In higher Neves–Zincke levels, the distal and proximal segments of the IVC, as well as the contralateral renal vein, were dissected and sequentially clamped in the following order: the IVC below the renal vein (distal segment), the contralateral renal vein, and finally the proximal IVC. After vascular clamping, the IVC wall was incised and the tumor thrombus was removed. Following thrombus extraction, the IVC was primarily closed using a double-layer continuous 4-0 vascular suture.

### 2.4. Statistical Analysis

Baseline characteristics are summarized as counts/percentages for categorical variables and as means ± standard deviation (SD) or medians with interquartile range (IQR) for continuous variables, as appropriate. Normality was assessed with the Shapiro–Wilk test. Between-group comparisons used the χ^2^ or Fisher’s exact test for categorical data and Student’s *t*-test or Mann–Whitney U test for continuous data, depending on distribution. Predictors of postoperative complications were evaluated using univariate logistic regression analysis, then statistically significant variables in the univariate model were used in multivariate analysis to find independent predictive factors of postoperative complications Survival was estimated by the Kaplan–Meier method and compared with the log-rank test. Predictors of overall survival were evaluated with univariable Cox proportional hazards models, then statistically significant variables in the univariate model were used in multivariate analysis to find independent predictive factors of OS. Two multivariate models were constructed. The first included both preoperative and postoperative variables, while the second was restricted to preoperative variables. The latter approach was used to avoid adjusting for postoperative complications and other variables that may arise as a consequence of surgery and may therefore not represent independent baseline prognostic factors. Receiver operating characteristic analysis for predictors of postoperative complications and OS were performed on statistically significant quantitative variables in the multivariate logistic regression model and multivariate Cox proportional hazard regression model, respectively. Unless otherwise stated, tests were two-sided with *p* < 0.05 considered statistically significant. Analyses were performed in PQStat (PQStat Software v.1.8.6.126, Poznań, Poland).

## 3. Results

A total of 235 patients were included in the analysis, with 123 patients classified as level 1 thrombus and 112 as level 2–4. The general clinical characteristics of the whole analyzed cohort are presented in Table 1.

## 4. General Patient Data

BMI was significantly lower in level 2–4 than in the level 1 group (26.73 vs. 25.31 kg/m^2^, *p* = 0.03). The NRS 2002 was significantly higher in the level 2–4 group compared to the level 1 group (4 vs. 3, *p* < 0.001). The CCI score was higher in the level 2–4 group than in the level 1 group; however, this difference was close to the statistical significance (*p* = 0.05). More tumors were localized in the left kidney in the level 1 group compared to the second group (53.57% vs. 32.52%, *p* = 0.001). The other features were comparable in both groups (*p* > 0.05) (Table 2).

### 4.1. Preoperative and Postoperative Laboratory Test Results

Pre- as well as postoperative hemoglobin level was significantly higher in the level 1 group compared to the level 2–4 group (12.21 vs. 11.15 g/dL, *p* < 0.001; 10.45 vs. 9.6 g/dL, *p* < 0.001, respectively). The other pre- and postoperative laboratory results were similar in both groups (*p* > 0.05) (Table 3).

### 4.2. Procedure Characteristics

The procedure duration was shorter in the level 1 group vs. level 2–4 group, however this difference was close to statistical significance (*p* = 0.06). Moreover, the blood loss was significantly lower in the level 1 group compared to the second group (264.11 mL vs. 599.18 mL, *p* = 0.001). Therefore, significantly more units of red blood cells were transfused in the level 2–4 group than in the level 1 group (0.69 vs. 1.46, *p* < 0.001). Hospitalization duration, as well as postoperative hospitalization duration, was significantly higher in the level 2–4 group than in the level 1 group (9 vs. 8, *p* < 0.001; 7 vs. 6 days, *p* < 0.001). In the level 2–4 group, significantly more postoperative complications were observed than in the level 1 group (35.37% vs. 13.39%), with hemorrhagic shock being most common in both groups which was more observed in the level 2–4 group compared to the level 1 group (26.06% vs. 9.82%, *p* = 0.001). Moreover, both compared VTT groups showed differences regarding complications graded according to the Clavien–Dindo classification (*p* < 0.001). Patients with VTT level 1 experienced significantly fewer major postoperative complications compared with those with VTT levels 2–4. Minor complications (Clavien–Dindo grades I–II) were observed in 0.89% of patients in the VTT 1 group, compared with 2.46% in the VTT 2–4 group. In contrast, major complications (grades III–V) occurred more than twice as frequently in the VTT 2–4 group than in the VTT 1 group (33.33% vs. 12.50%). This difference was statistically significant (*p* < 0.001).

Notably, severe complications were driven primarily by a higher incidence of grade IV events in the VTT 2–4 cohort, particularly grade IVa (21.95% vs. 8.93%) and grade IVb (7.3% vs. 1.8%). Postoperative mortality (grade V) was also numerically higher in patients with more advanced thrombus extension (4.1% vs. 1.8%), although the absolute number of events was low. These findings indicate that increasing VTT level is associated with a substantially greater risk of severe postoperative morbidity and mortality (Table 4).

### 4.3. Predictive Factors of Postoperative Complications

In univariate logistic regression analysis, the NRS 2002 (Odds Ratio (OR) = 4.15, *p* < 0.001), level 2–4 of tumor vein invasion (OR = 3.73, *p* < 0.001), procedure time (OR = 1.02, *p* = 0.001), blood loss (OR = 1.002, *p* < 0.001), preoperative hemoglobin (OR = 0.79, *p* = 0.001), preoperative white blood cells (OR = 1.08, *p* = 0.03), preoperative lymphocytes (OR = 0.82, *p* = 0.008), preoperative neutrophils (OR = 1.19, *p* < 0.001), MLR (OR = 4.86, *p* = 0.001), NLR (OR = 1.18, *p* < 0.001) and PLR (OR = 1.002, *p* = 0.005) met the *p* < 0.05 criterion and were analyzed in the multivariate logistic regression model (Table 5).

**Figure 1:**
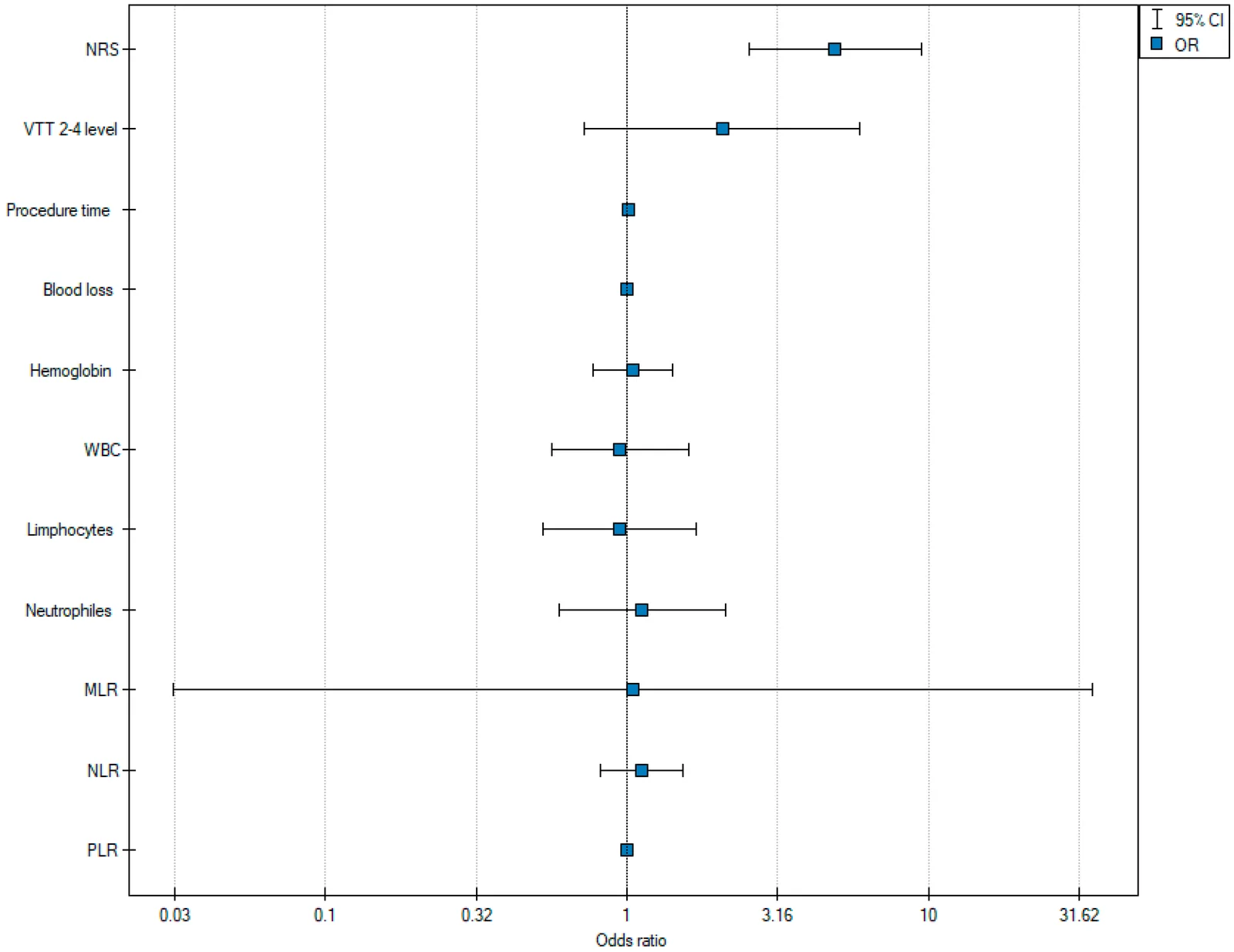
Multivariate logistic regression analysis of predictive factors for postoperative complications. Abbreviations: MLR—Monocytes-Lymphocytes ratio, NLR—Neutrophils-Lymphocytes ratio, NRS—Nutritional risk score, PLR—Platelets-Lymphocytes ratio, WBC—White blood cell count, VTT—Venous tumor thrombus.

In the multivariate model, the NRS 2002 (OR = 4.90, 95% CI = 2.53–9.48, *p* < 0.001) and blood loss (OR = 1.002, 95% CI = 1.001–1.003; *p* < 0.001) proved to be independent predictive factors for postoperative complications (Figure 1).

**Figure 2:**
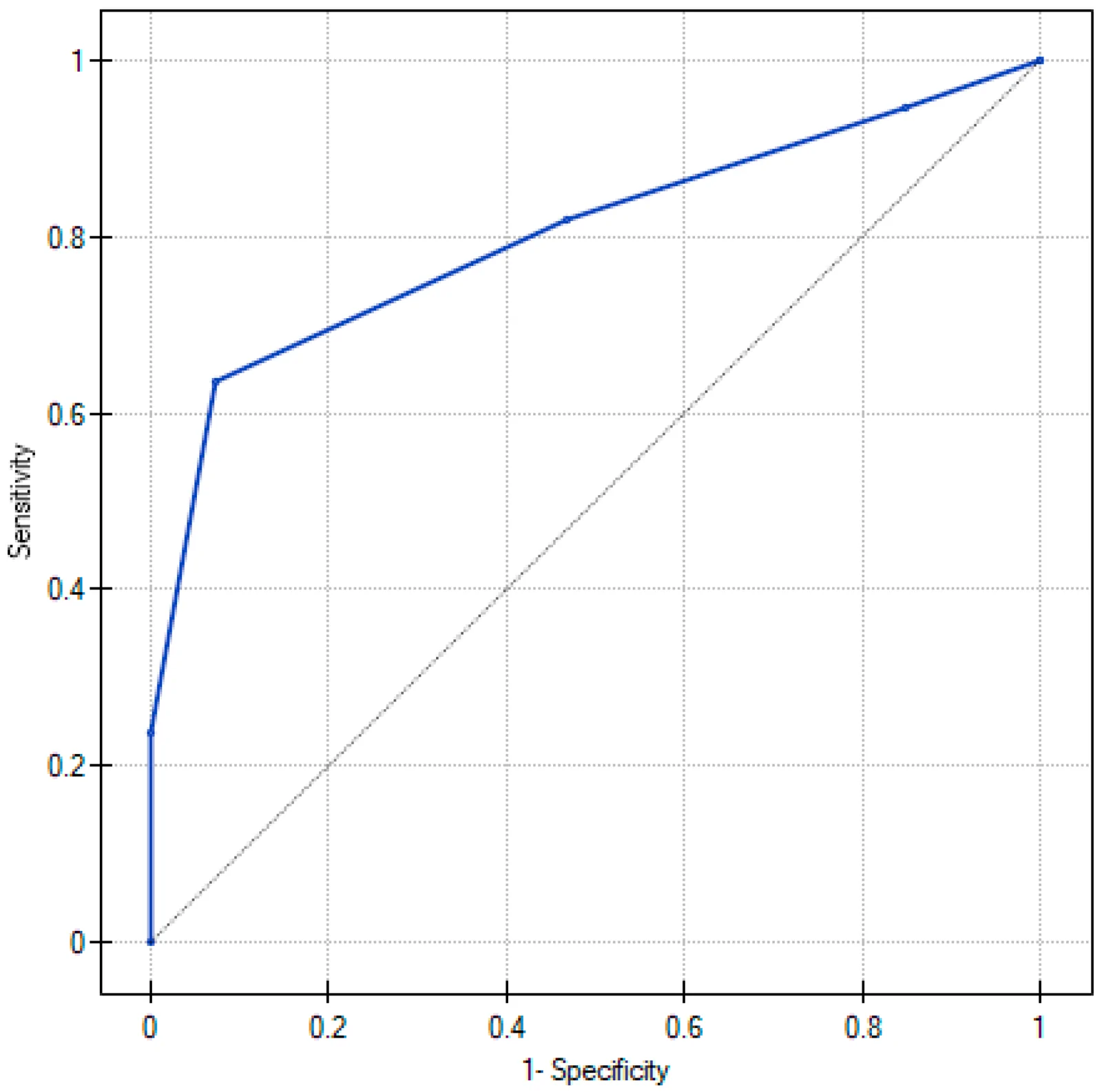
Receiver operating characteristic analysis of NRS as a predictor of postoperative complications. Footnote: AUC = 80.22%, SE = 3.89%, *p* < 0.001, cut-off point ≥ 5 points.

The ROC analysis was performed for NRS 2002 and blood loss. In the analysis of ROC of NRS, the AUC = 80.22%, SE = 3.89%, *p* < 0.001, cut-off point ≥ 5 points (Figure 2).

**Figure 3:**
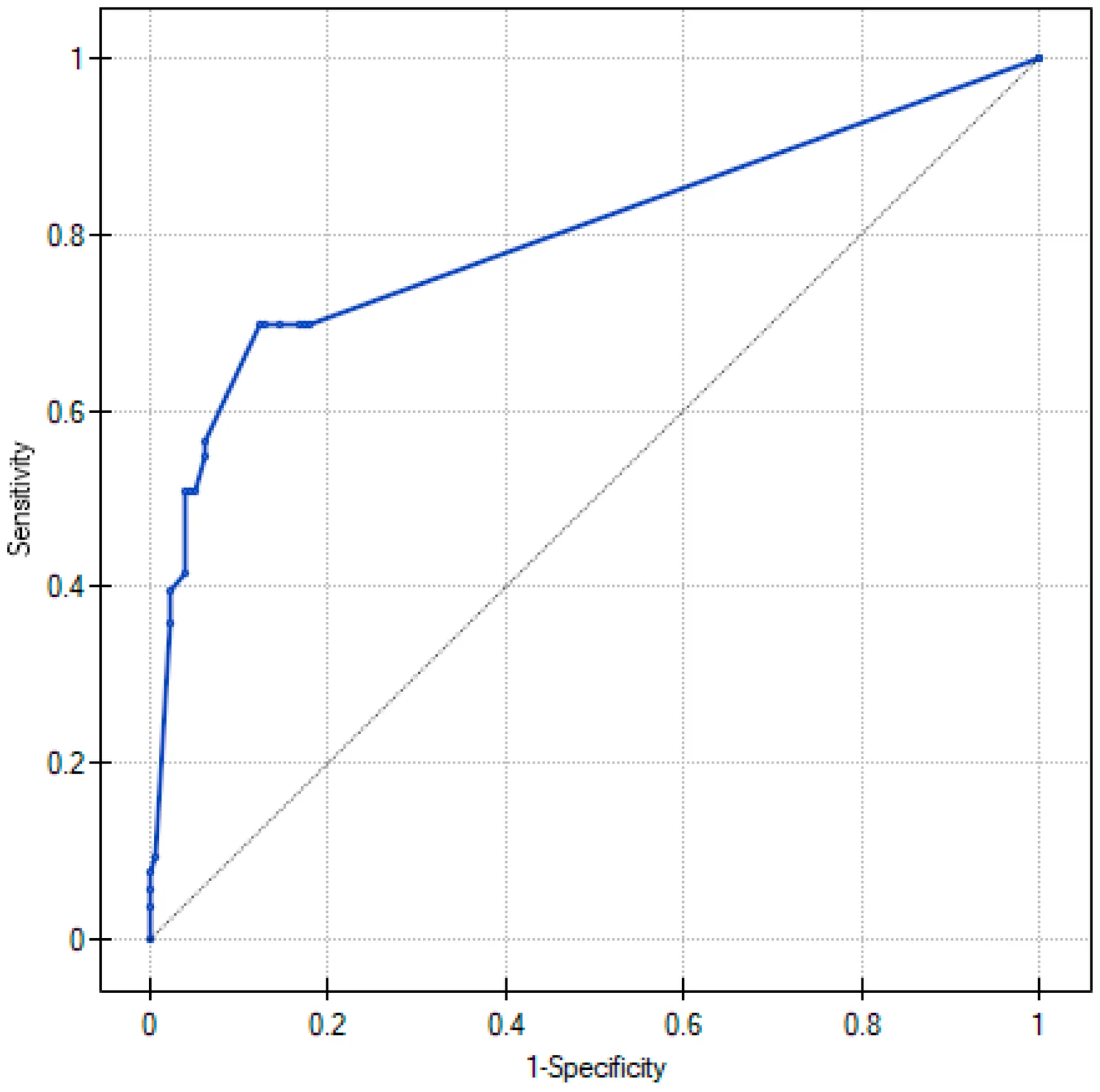
Receiver operating characteristic analysis of blood loss as a predictor of postoperative complications. Footnotes: AUC= 79.77%, SE = 3.69%, *p* < 0.001, cut-off point = 1000 mL.

Moreover, in the ROC analysis of blood loss, the AUC= 79.77%, SE = 3.69%, *p* < 0.001, cut-off point = 1000 mL (Figure 3).

### 4.4. Tumor Characteristics

The tumor size was significantly larger in the level 2–4 group compared to the level 1 group (8.75 vs. 7.5 *p* < 0.001). There was no difference regarding histopathological type between the two groups (*p* > 0.05) (Table 6).

### 4.5. Follow-Up

The median follow-up time was 22 months (0–159, IQR 42.5 months). The median OS was 45 months. The 1-year OS was 71.1%, while the 5-year OS was 42.49%.

**Figure 4:**
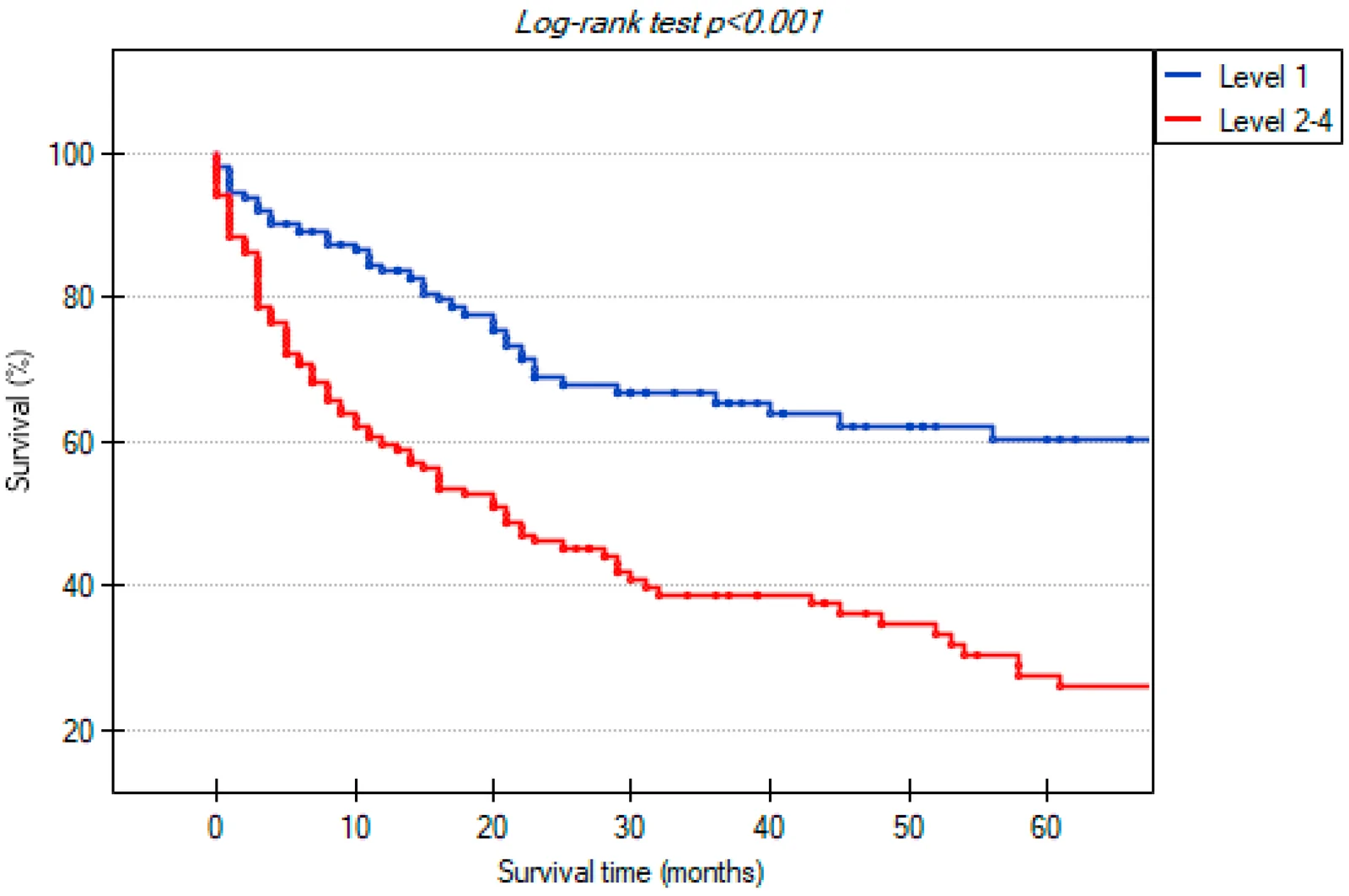
Kaplan–Meier overall survival curves. Footnote: Level 1 vs. level 2–4 1-year overall survival (83.61% SE 3.53% vs. 59.65% SE 4.44%, *p* < 0.001). Level 1 vs. level 2–4 5-year overall survival (60.24% SE 5.29% vs. 27.41% SE 4.73%, *p* < 0.001).

The median follow-up duration was longer for the level 1 group than for the level 2–4 group (30 vs. 16, *p* = 0.001). This may be associated with lower 1- and 5-year overall survival in the level 2–4 group than in the level 1 group (59.65% vs. 83.61%, 27.41% vs. 60.24%, both *p* < 0.001) (Table 7) (Figure 4).

Two multivariate models were constructed. The first included both preoperative and postoperative variables, while the second was restricted to preoperative variables. The latter approach was used to avoid adjusting for postoperative complications and other variables that may arise as a consequence of surgery and may therefore not represent independent baseline prognostic factors.

In the univariate Cox proportional hazard regression analysis (first model including both preoperative and postoperative variables), the BMI (Hazard ratio (HR) = 0.94, *p* = 0.01), NRS 2002 (HR = 1.38, *p* < 0.001), CCI score (HR = 1.22, *p* < 0.001), Level 2–4 of tumor vein invasion (HR = 2.40, *p* < 0.001), blood loss (HR = 1.0004, *p* < 0.001), preoperative hemoglobin (HR = 0.77, *p* < 0.001), preoperative monocytes (HR = 1.20, *p* = 0.04), preoperative platelets (HR = 1.003, *p* < 0.001), preoperative PLR (HR = 1.002, *p* = 0.001), preoperative sodium (HR = 0.93, *p* < 0.001), preoperative potassium (HR = 1.88, *p* < 0.001), postoperative hemoglobin (HR = 0.75, *p* < 0.001), postoperative white blood cells (HR = 1.02, *p* = 0.04), postoperative monocytes (HR = 1.51, *p* = 0.045), postoperative platelets (HR = 1.003, *p* < 0.001), postoperative sodium (HR = 0.91, *p* < 0.001), postoperative potassium (HR = 1.58, *p* < 0.001), occurrence of postoperative complications (HR = 2.26, *p* < 0.001), tumor size (HR = 1.09, *p* < 0.001), T4 (HR = 1.82, *p* = 0.002), N1-2 (HR = 1.58, *p* = 0.002), and M1 (HR = 2.58, *p* = 0.01) met the *p* < 0.05 criterion and were analyzed in the multivariate model (Table 8).

**Figure 5:**
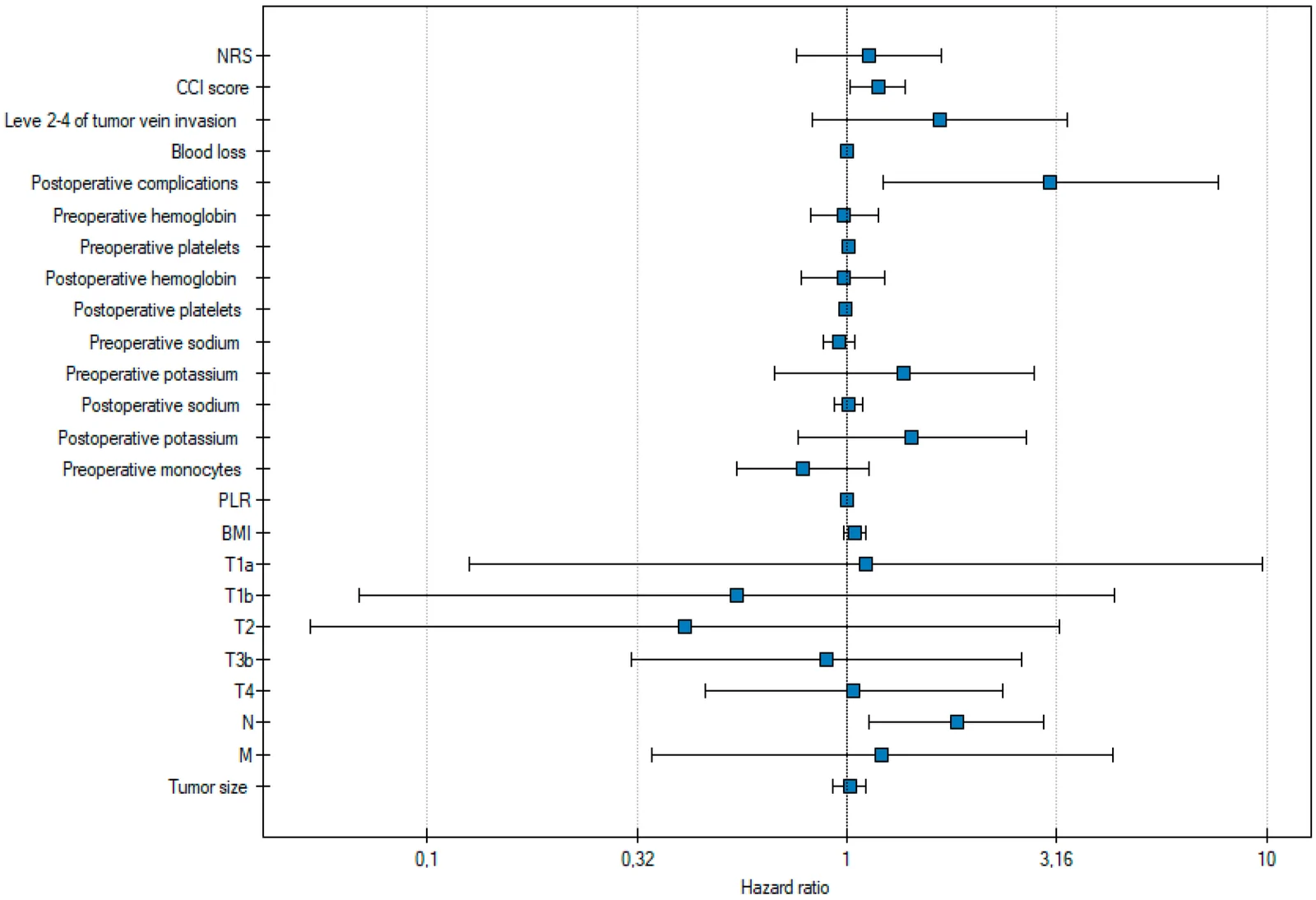
Multivariate Cox proportional hazard regression analysis of predictive factors of overall survival. Footnote: CCI score (HR = 1.19, 95% CI = 1.02–1.38, *p* = 0.03); occurrence of postoperative complications (HR = 3.06, 95% CI = 1.22–7.67, *p* = 0.01), preoperative platelets (HR = 1.01, 95% CI = 1.001–1.01, *p* = 0.02), postoperative platelets (HR = 0.99, 95% CI = 0.988–0.99, *p* = 0.04), N1-2 (HR = 1.83, 95% CI = 1.13–2.94, *p* = 0.01).

In multivariate Cox proportional hazard regression analysis, the CCI score (HR = 1/19, 95% CI = 1.02–1.38, *p* = 0.03), occurrence of postoperative complications (HR = 3.06, 95% CI = 1.22–7.67, *p* = 0.01), preoperative platelet count (HR = 1.01, 95% CI = 1.001–1.01, *p* = 0.02), postoperative platelet count (HR = 0.99, 95% CI = 0.988–0.99, *p* = 0.04), and N1-2 (HR = 1.83, 95% CI = 1.13–2.94, *p* = 0.01) were independent predictive factors of OS (Figure 5).

**Figure 6:**
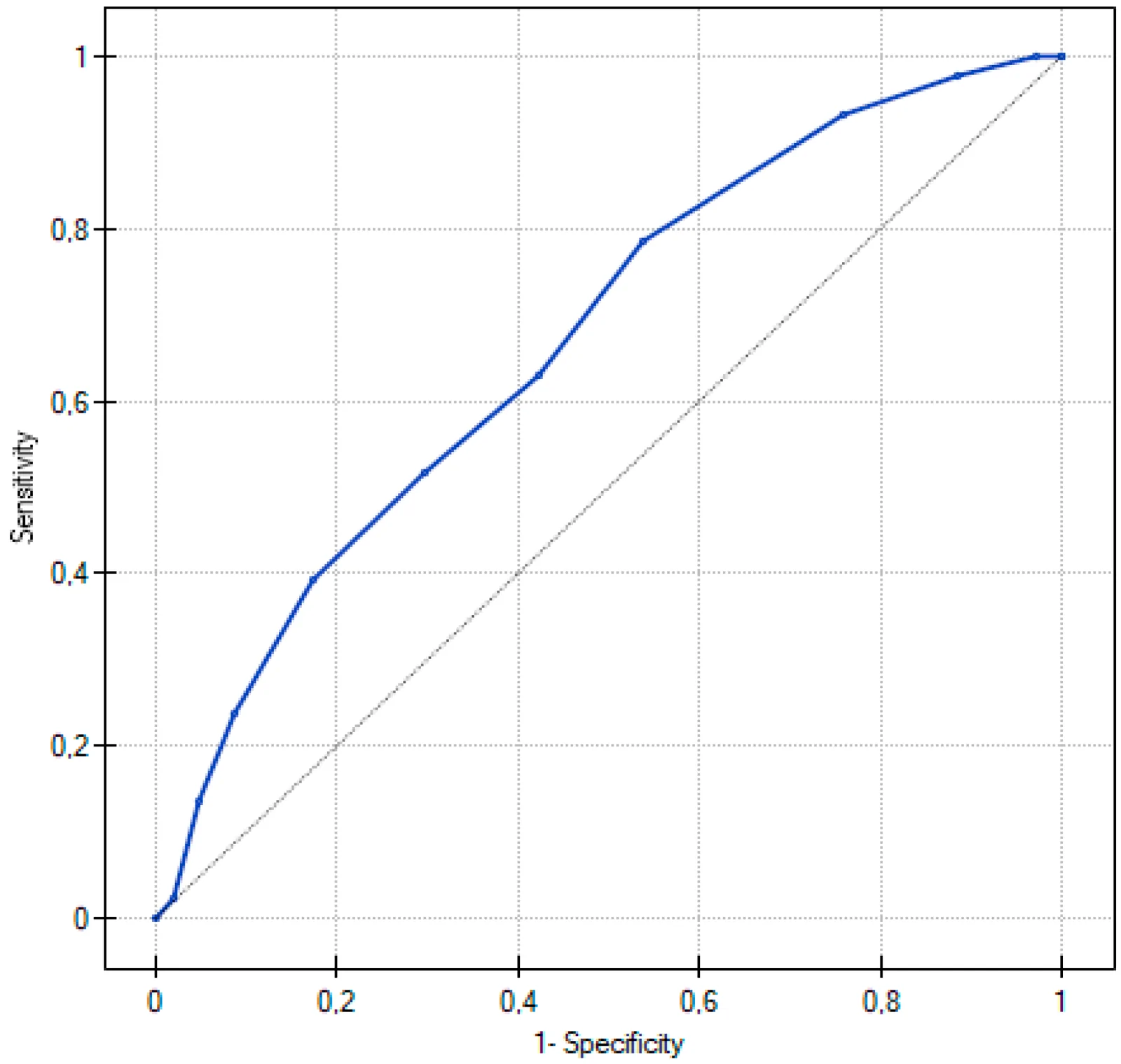
Receiver operating characteristic analysis of CCI score for predicting the overall survival. Footnote: AUC = 67.09%, SE = 3.82%, *p* < 0.001; cut-off point ≥ 6 points.

ROC analysis was performed on statistically significant quantitative variables in the multivariate Cox proportional hazard regression model. In ROC analysis of CCI score, the AUC = 67.09%, SE = 3.82%, *p* < 0.001 and cut-off point ≥ 6 points (Figure 6).

**Figure 7:**
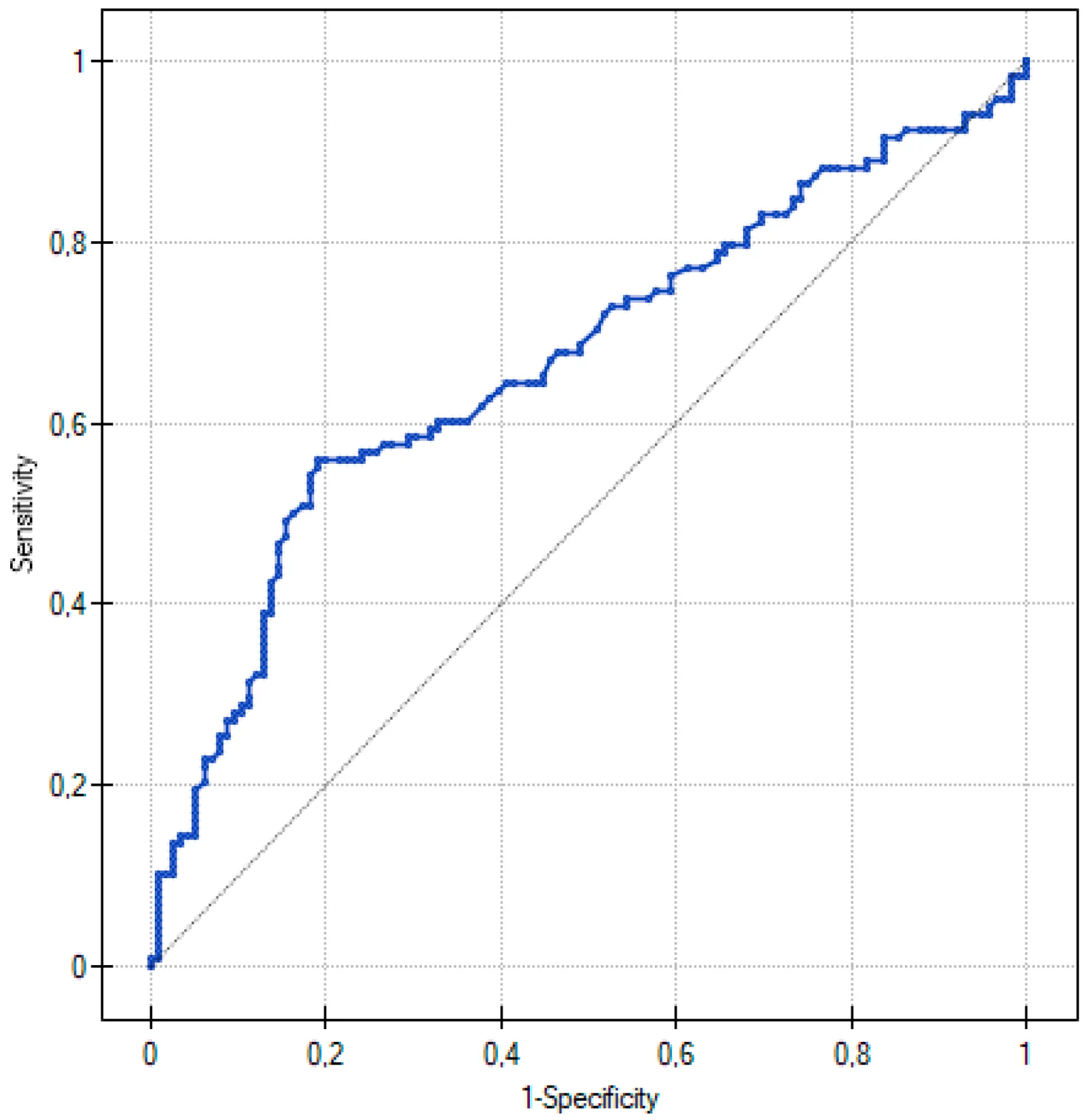
Receiver operating characteristic analysis of preoperative platelets for predicting the overall survival. Footnote: AUC = 66.76%, SE = 3.58%, *p* < 0.001 cut-off point ≥ 302 G/L.

In ROC analysis of preoperative platelet count, the AUC = 66.76%, SE = 3.58%, *p* < 0.001 and cut-off point ≥ 302 G/L (Figure 7).

**Figure 8:**
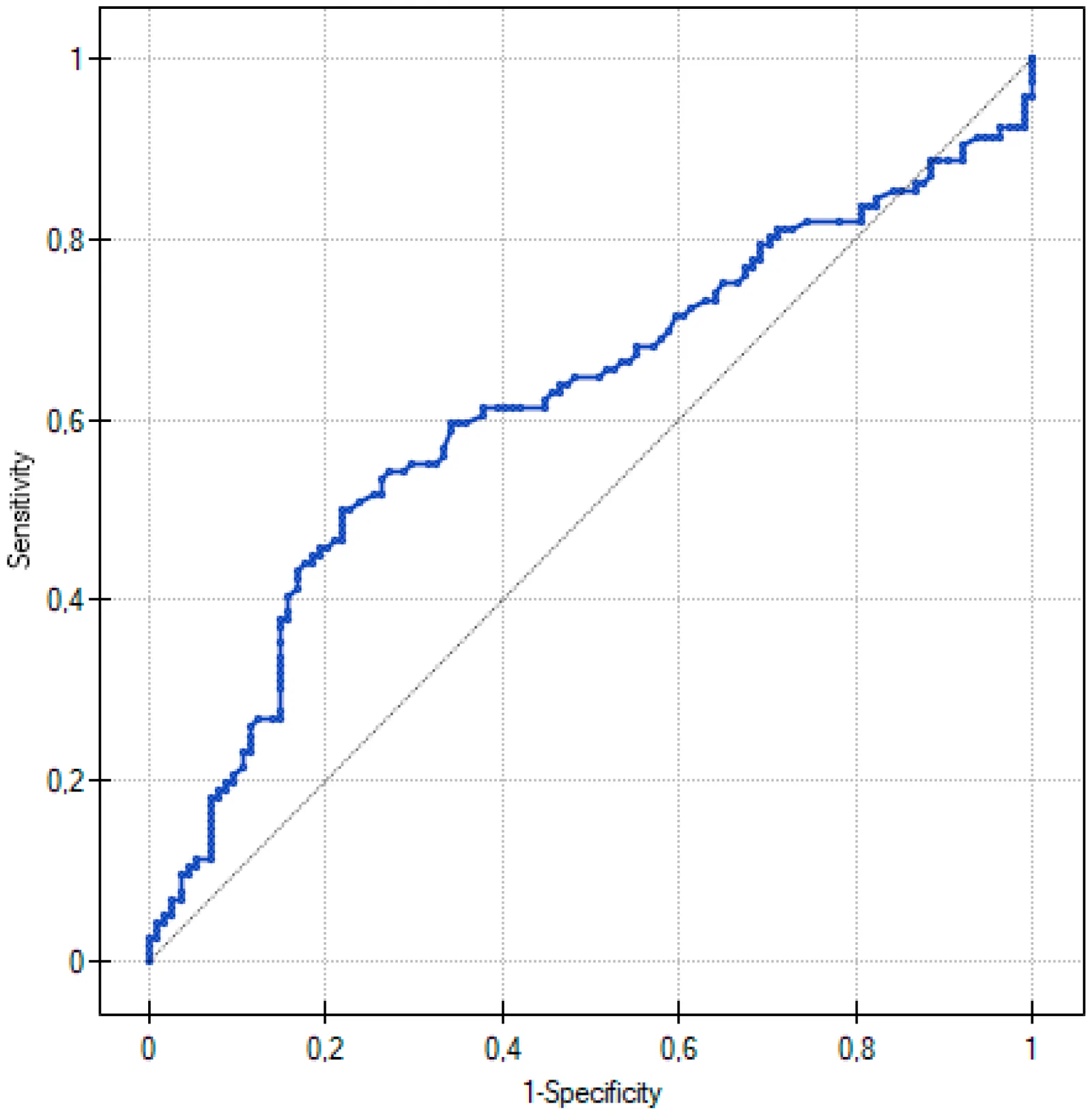
Receiver operating characteristic analysis of postoperative platelets for predicting the overall survival. Footnote: AUC = 61.78%, SE = 3.76%, *p* < 0.001, cut-off point ≥ 244 G/L.

In ROC analysis of postoperative platelet count, the AUC = 61.78%, SE = 3.76%, *p* < 0.001 and cut-off point ≥ 244 G/L (Figure 8).

In the univariate Cox proportional hazard regression analysis (second model restricted to preoperative variables), the BMI (Hazard ratio (HR) = 0.94, *p* = 0.01), NRS 2002 (HR = 1.38, *p* < 0.001), CCI score (HR = 1.22, *p* < 0.001), Level 2–4 of tumor vein invasion (HR = 2.40, *p* < 0.001), blood loss (HR = 1.0004, *p* < 0.001), preoperative hemoglobin (HR = 0.77, *p* < 0.001), preoperative monocytes (HR = 1.20, *p* = 0.04), preoperative platelets (HR = 1.003, *p* < 0.001), preoperative PLR (HR = 1.002, *p* = 0.001), preoperative sodium (HR = 0.93, *p* < 0.001), preoperative potassium (HR = 1.88, *p* <0.001), tumor size (HR = 1.09, *p* < 0.001), T4 (HR = 1.82, *p* = 0.002), N1-2 (HR = 1.58, *p* = 0.002), and M1 (HR = 2.58, *p* = 0.01) met the *p* < 0.05 criterion and were analyzed in the multivariate model (Table 9).

**Figure 9:**
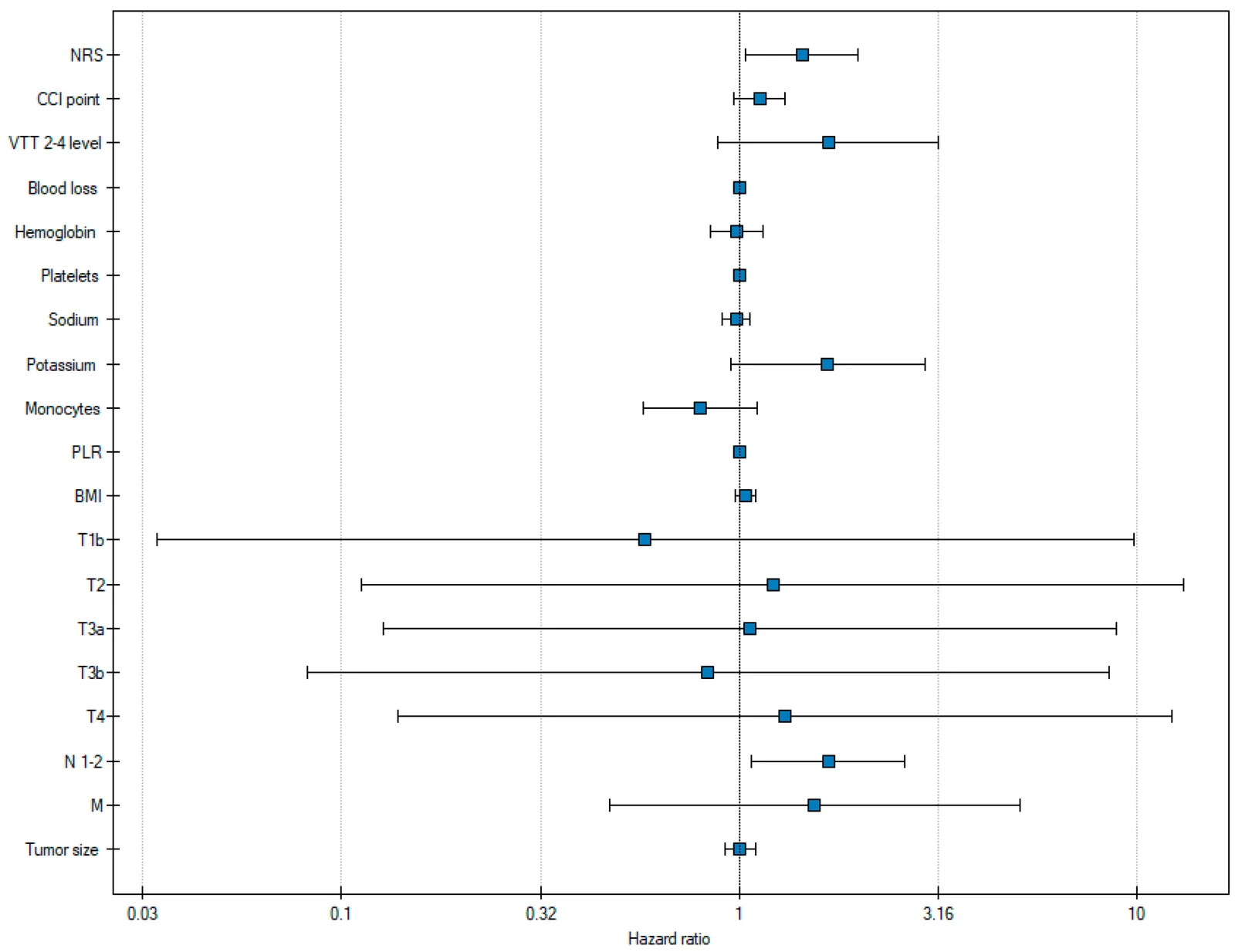
Multivariate Cox proportional hazard regression analysis of predictive factors of overall survival. Footnote: The NRS 2002 (HR = 1.44, 95% CI = 1.04–1.99, *p* = 0.03), N1-2 (HR = 1.67, 95% CI = 1.07–2.61, *p* = 0.02).

In multivariate Cox proportional hazard regression analysis, the NRS 2002 (HR = 1.44, 95% CI = 1.04–1.99, *p* = 0.03) and N1-2 (HR = 1.67, 95% CI = 1.07–2.61, *p* = 0.02) were independent predictive factors of OS (Figure 9).

**Figure 10:**
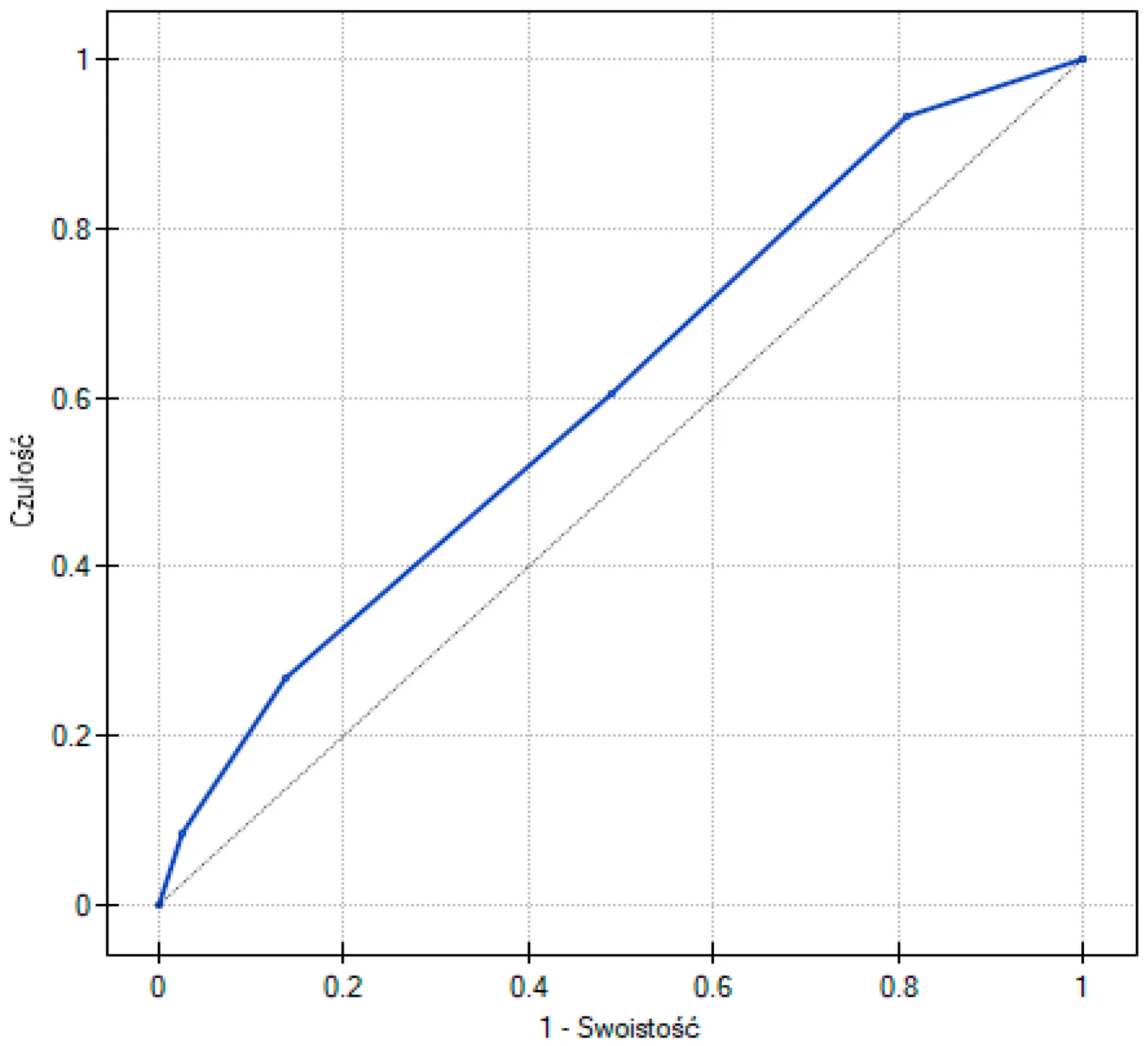
Receiver operating characteristic analysis of NRS for predicting the overall survival. Footnote: The AUC = 60.39%, SE = 3.51%, *p* < 0.001 and cut-off point ≥ 5 points.

ROC analysis was performed on statistically significant quantitative variables in the multivariate Cox proportional hazard regression model. In ROC analysis of NRS 2002, the AUC = 60.39%, SE = 3.51%, *p* < 0.001 and cut-off point ≥ 5 points (Figure 10).

## 5. Discussion

Our study provides a comprehensive evaluation of perioperative and oncological outcomes in patients with RCC-VTT, with particular emphasis on nutritional status and perioperative factors. Our findings are largely consistent with the existing literature, while also contributing novel insights into contemporary risk stratification.

Consistent with prior reports, in our study, higher thrombus levels (2–4) were associated with increased surgical burden, including greater intraoperative blood loss, higher transfusion rates, and more frequent postoperative complications as well as prolonged duration of hospitalization. These observations align with established data demonstrating that surgical complexity escalates with cranial thrombus extension [1,5,13,14,15]. The study by Blute et al. (2004) [6] showed significantly more early complications in patients with a higher thrombus level (respectively for level 0 to IV, 8.6%, 15.2%, 14.1%, 17.9% and 30.0%, *p* < 0.001). In this study, there was a statistically significant difference in outcome for patients with level 0 (RV) versus those with level > 0 (IVC) thrombus (*p* = 0.002), but there was no significant difference in outcome by thrombus level among patients with IVC tumor thrombus (*p* = 0.868) [6]. These findings were supported by more recent multi-center analyses [13,14] suggesting that higher thrombus levels (2–4) were associated with significantly increased intraoperative blood loss, greater transfusion requirements, and prolonged hospitalization. Similarly, the incidence of postoperative complications was significantly higher in patients with advanced thrombus extension, which is consistent with most contemporary series and systematic reviews [13,15]. In a study by Abel et al. [13] including 162 patients, a multivariate analysis demonstrated that level 4 thrombus was independently associated with increased risk of major complications defined as ≥3a according to the Clavien–Dindo classification [13]. In a study by Chen et al. [15] including 121 patients, a longer median operative time (438 min vs., 311 min, *p* < 0.001), greater median blood loss (2550 mL vs. 500 mL, *p* < 0.001), and a longer duration of hospitalization (12.5 days vs. 9 days, *p* = 0.004) in higher VTT level (Mayo III–IV) compared to lower VTT level (Mayo 0-II) were reported [15]. Nagamoto et al. [16] showed the positive correlation between VTT level and operation time (*p* < 0.001) and blood loss (*p* < 0.001) [16]. Faria-Costa et al. [17] also reported the significant association between VTT level and operative time, intraoperative blood loss, and duration of hospitalization (*p* < 0.05) [17]. In a study by Dell’Oglio et al. [18], although there were no statistically significant differences between patients with level III–IV and level I–II thrombus regarding duration of hospitalization (14 vs. 12 days), in patients with level III–IV thrombus, a significantly higher incidence of postoperative transfusions and postoperative complications compared to those with level I–II thrombus (82% vs. 33%, *p* < 0.05) was noted. Severe complications classified as Clavien–Dindo grade ≥ 3 occurred significantly more frequently in the level III–IV group (41% vs. 15%, *p* < 0.001) [18]. Another study by Navratil et al. [19] revealed longer duration of operation in patients with higher VTT, and differences in complication rate regarding VTT level [19]. In a study by Lee et al. [20], higher VTT level was associated with more perioperative complications [20]. Overall, our results, as well all findings mentioned above, reinforce that thrombus extent remains a key determinant of surgical difficulty.

In the multivariable analysis based on preoperative variables, OS was primarily associated with patient-related and disease-related factors, including NRS 2002 and lymph node involvement. In a separate model incorporating both preoperative and postoperative variables, postoperative complications were independently associated with OS and were associated with a more than threefold increased risk of mortality. However, because of postoperative complications, and postoperative hemoglobin and platelet counts occur after surgery, their associations with OS should be interpreted with caution. These variables may reflect the consequences of the surgical procedure and postoperative course rather than baseline prognostic characteristics. Therefore, the association between postoperative complications and long-term survival should be regarded as an observation of prognostic relevance rather than evidence of a causal effect. Nevertheless, the observed association is clinically important and is consistent with previous studies in oncological surgery reporting relationships between postoperative morbidity and long-term outcomes [21,22,23]. In the context of RCC-VTT, these findings highlight the importance of careful preoperative assessment and perioperative management, while emphasizing that the prognostic role of postoperative variables requires cautious interpretation.

Regarding the impact of other factors on survival, similarly to our observations, recent evidence, including the meta-analysis by Lasorsa et al. [24] and newer large-scale analyses [15,16], suggests that tumor-related and patient-related characteristics are associated with outcomes in patients with RCC-VTT, beyond the anatomical extent of venous tumor thrombus. Our findings similarly indicate that VTT level alone may have limited prognostic value after adjustment for other clinical and pathological variables. However, given the retrospective design of our study, these associations should not be interpreted as causal. Rather, they support the consideration of an integrated prognostic approach that takes into account both tumor-related and patient-related characteristics when assessing outcomes in patients with RCC-VTT.

Only several authors noted the association between higher VTT level and worse prognosis. Bokka et al. reported the association between VTT level and worse median survival [25]. Similarly, worse OS in higher VTT level (III–IV compared to I–II) was reported by Dell’Oglio et al. [18], and worse DFS in higher VTT was noted by Navratil et al. [19].

According to most authors, including Chen et al. [15], Lee et al. [20], Shiff et al. [26], Hanquiez et al. [27], and Miura et al. [28], VTT level is not an independent predictor for survival in patients with RCC-VTT. In the Faria-Costa et al. study [17], tumor biology is a stronger predictor for survival than VTT level [17]. Wagner et al. [29] reported that the level of tumor thrombus in the IVC did not significantly affect long-term OS in patients with RCC. The OS was statistically different in patients with VTT located in the RV compared to those with IVC involvement [29].

Overall, the association between VTT level and survival is controversial [30]. Most recent studies have observed that there was no association between thrombus level and prognosis [15,20,26,27,28,30,31,32,33]. In contrast, others, mainly earlier studies, have reported differences in prognosis between patients with a tumor thrombus extending below the diaphragm (or hepatic vein) and those extending above the diaphragm (hepatic vein) [18,19,25,34,35,36], and some authors have noted that the cut-line for predicting prognosis differed between patients with a tumor thrombus within the RV and those with a thrombus extending into the IVC [6,29]. Thus, our results contribute to the ongoing debate regarding the prognostic significance of thrombus level. In our opinion, the above-mentioned contradictory findings are associated with substantial heterogeneity in patient populations, inclusion criteria, tumor biology, surgical management, and follow-up duration. In addition, VTT level was not a significant prognostic factor in multivariate analyses, which typically included more comprehensive pathological and clinical covariates. In contrast, studies that reported a statistically significant association may have relied solely on univariate analyses or may have failed to adequately adjust for key factors, such as tumor biology.

Furthermore, the association between intraoperative blood loss and adverse outcomes in our cohort is consistent with previous reports linking greater surgical burden with increased morbidity and impaired recovery [12,37,38,39]. The meta-analysis by Liu et al. [38], including 10 retrospective studies and 19,240 patients, found that perioperative blood transfusion was associated with significantly worse overall survival (OS; HR 2.62, 95% CI: 1.98–3.46), recurrence-free survival (RFS; HR 2.55, 95% CI: 1.74–3.75), and cancer-specific survival (CSS; HR 3.15, 95% CI: 2.30–4.31). However, the authors highlighted potential confounding, particularly differences in tumor stage among the included studies. In our cohort, blood loss ≥ 1000 mL was associated with postoperative complications and remained an independent predictor in multivariate analysis, whereas its association with OS was observed only in univariate analysis. These findings suggest that blood loss may reflect surgical complexity and disease burden rather than represent an independent causal determinant of long-term survival. Moreover, the identification of a clinically relevant threshold (≥1000 mL) in our study may provide a practical intraoperative benchmark for risk stratification and perioperative decision-making.

Importantly, our study highlights the role of patient-related factors, particularly nutritional status, which remains underrepresented in the existing literature. A particularly novel and clinically relevant aspect of our study is the demonstrated role of nutritional status assessed using the NRS 2002. Unlike inflammatory markers such as NLR, which did not retain significance in multivariate analysis, NRS 2002 proved to be a strong independent predictor of postoperative complications (OR = 5.11, 95% CI = 2.62–9.97, *p* < 0.001) in our study. In the ROC analysis of NRS 2002, the AUC = 80.24%, SE = 3.69%, *p* < 0.001 and cut-off point = 5 points was reported. Moreover, NRS 2002 is also a significant prognostic factor for OS in the univariate Cox regression analysis (HR 1.38, *p* < 0.001) in our patients. This finding is in line with the broader oncological framework proposed by McMillan et al. [40], emphasizing the importance of systemic inflammation and nutritional status in cancer outcomes. Importantly, our study extends these concepts specifically to the RCC-VTT population, where such data have been scarce [40]. The relatively high discriminative ability of NRS 2002 (AUC 80.24%) in our study further underscores its potential clinical utility. Compared to isolated laboratory markers, NRS 2002 provides a more comprehensive assessment of patient frailty by integrating both nutritional impairment and disease severity, which may explain its superior predictive performance [11]. Only one study regarding the role of NRS 2002 in RCC has been published, but the role of NRS 2002 in patients with RCC-VTT, reported in our study, is novel [41,42]. Thus, this finding remains underrepresented in the existing literature. While prior studies have focused predominantly on tumor characteristics and inflammatory markers such as NLR and its association with survival [16], our results demonstrate that NRS 2002 provides an independent and stronger predictive value for postoperative complications. This suggests that comprehensive assessment of patient frailty may offer superior risk stratification compared to isolated laboratory-based indices.

Pre- as well as postoperative hemoglobin levels were significantly higher in the level 1 group compared to the level 2–4 group. Additionally, preoperative and postoperative hemoglobin were significant predictive factors for postoperative complications. In addition, preoperative and postoperative hemoglobin are significant predictors of OS in the univariate Cox regression analysis in multivariate analysis including both preoperative and postoperative variables. Significant difference in hemoglobin concentrations between lower (level 1) and higher (levels 2–4) VTT suggest a relationship between more advanced disease and anemia, which is commonly observed in patients with RCC. Potential mechanisms include systemic chronic inflammation, impaired erythropoietin production, and increased tumor burden. Our findings are in line with the existing literature indicating that anemia may reflect more aggressive disease biology and has been associated with worse clinical outcomes in RCC [43,44]. According to Mussalam et al. [44], preoperative anemia, even to a mild degree, is independently associated with an increased risk of 30-day morbidity and mortality in patients undergoing major non-cardiac surgery. This finding further supports the concept that host-related factors play a central role in shaping both perioperative and oncological results.

An additional noteworthy finding is the prognostic significance of platelet count. In our patients, elevated preoperative and postoperative platelet levels were independently associated with worse survival in the univariate and multivariate Cox proportional hazard regression analysis, respectively. This finding may reflect tumor-associated systemic inflammation and prothrombotic activity. Recent studies (Uetani et al., Xiao et al.) have increasingly recognized systematic inflammatory markers, including thrombocytosis, as a negative prognostic marker in RCC and other malignancies [45,46], although its role in RCC-VTT remains insufficiently defined. However, given the postoperative timing of platelet assessment, this finding should be interpreted cautiously and should not be considered evidence of an independent baseline prognostic effect. Overall, our findings support further investigation of hematological parameters as potential prognostic markers in RCC-VTT, particularly when assessed before surgery.

The incorporation of systemic and potentially modifiable parameters, including hemoglobin level, platelet count, and nutritional status assessed by NRS 2002 is a key novel aspect of our study. These variables, reflecting the patient’s inflammatory, hematologic, and metabolic condition, were found to have significant prognostic implications yet remain underrepresented in existing RCC-VTT nomograms [47,48,49,50,51,52,53,54]. Their inclusion highlights the importance of host-related factors in cancer progression and surgical outcomes, moving beyond traditional tumor-centered models.

Therefore, our findings may serve as a basis for future development of an integrated prognostic nomogram integrating both tumor-related and patient-related variables, including VTT level, CCI, lymph node status, postoperative complications, as well as Hb, PLT, and NRS 2002. In contrast to previously published models [47,48,49,50,51,52,53,54], which predominantly focus on pathological and anatomical features, our approach incorporates modifiable systemic parameters that may be actively optimized. While existing nomograms [47,48,49,50,51,52,53,54] provide valuable risk stratification, they do not fully capture the impact of perioperative condition and host-related factors. Therefore, our model may offer improved clinical utility by combining prognostic accuracy with actionable therapeutic implications.

Importantly, several of the identified prognostic factors, such as anemia, thrombocytosis, and malnutrition, are potentially modifiable. This creates an opportunity for targeted prehabilitation strategies aimed at optimizing the patient’s condition prior to surgery. Interventions such as nutritional support, correction of anemia, and management of systemic inflammation may not only reduce perioperative complications but also improve long-term outcomes. Thus, the proposed model may serve not only as a prognostic tool but also as a framework for individualized preoperative optimization.

Overall, our findings support a comprehensive approach to the management of RCC-VTT, in which anatomical tumor extent is considered alongside patient- and disease-related characteristics and the substantial perioperative complexity of surgical treatment [55]. Further prospective studies are warranted to validate these associations and to better define the prognostic role of preoperative and postoperative factors in this population.

## 6. Limitations

This study has several limitations. First, it is a single-center, retrospective analysis, which may introduce selection bias. Second, although all procedures were performed by experienced surgeons, variability in surgical techniques and perioperative management may have contributed to heterogeneity in outcomes. Third, despite the inclusion of multiple clinically relevant variables, detailed molecular and genetic tumor characteristics were not available. Additionally, the potential impact of systemic therapies on survival outcomes was not fully captured. Finally, changes in surgical techniques and perioperative care over the study period may have influenced the results. Therefore, prospective, multi-center studies with external validation are warranted to confirm our findings.

## 7. Conclusions

An important finding of the present study is that, although higher VTT levels (VTT 2–4) were associated with greater surgical complexity, increased blood loss, and a higher rate of perioperative complications, VTT level was not an independent predictor of OS. These findings suggest that thrombus level alone may have limited prognostic value when considered together with other clinical and pathological factors. However, the present study cannot determine whether VTT level primarily reflects surgical complexity, tumor biology, or both. Therefore, prognostic assessment of patients with RCC-VTT should consider a broader range of patient- and disease-related characteristics rather than anatomical thrombus extent alone.

Postoperative complications were associated with a substantially increased risk of mortality in the multivariable model including postoperative variables. However, given the retrospective design and the postoperative nature of this variable, this association should be interpreted cautiously and does not establish a causal relationship between complications and long-term survival. Nevertheless, the finding emphasizes the clinical importance of perioperative management and warrants further investigation in prospective studies.

In conclusion, higher VTT levels were associated with greater surgical complexity and perioperative morbidity but were not independently associated with long-term survival. In the analysis based on preoperative variables, OS was associated with patient- and disease-related factors, including nutritional status classified according to NRS 2002 and lymph node involvement. The associations observed for postoperative complications and hematological parameters should be interpreted with caution, as these variables may reflect the postoperative course rather than baseline prognostic characteristics. Overall, our findings support a comprehensive approach to risk assessment in patients with RCC-VTT, integrating anatomical, tumor-related, and patient-related factors, while highlighting the need for further prospective studies to validate these associations.

## Figures and Tables

**Table 1:** General characteristics of the study group.

Variable	*n* (%), Mean/Median (Min–Max) SD/IQR
Age (years)	65.45 (30–87) SD 10.66
Gender	
Male	152 (64.68%)
Female	83 (35.32%)
BMI	25.87 (18.03–49.27) IQR 6.28
Tumor side	
Right	135 (57.44%)
Left	100 (42.55%)
Tumor diameter (cm)	8 (0.2–24) IQR 4
Hospital stay (days)	9 (2–39) IQR 4
Operative time (min)	115 (40–285) IQR 55
Blood loss volume (ml)	442.25 (0–4000) SD 797.74
Blood transfusion volume (units)	1.09 (0–10) SD 1.67
Neves–Zincke classification	
1	112 (47.66%)
2	73 (31.06%)
3	48 (20.43%)
4	2 (0.85%)
Surgery type	
Nephrectomy	230 (97.87%)
Laparotomy	5 (2.12%)
Adrenalectomy	156 (66.38%)
Lymphadectomy	55 (23.40%)
TNM classification	
T	
1a	4 (1.70%)
1b	11 (4.68%)
2a	13 (5.53%)
3a	149 (63.40%)
3b	11 (4.68%)
3c	1 (0.43%)
4	22 (9.36%)
N	
0	204 (86.81%)
1	23 (9.79%)
2	8 (3.40%)
M	
0	225 (95.75%)
1	10 (4.26%)
Histopathological type	
Fuscocellular + Clear cell	1 (0.43%)
Fuscocellular	2 (0.85%)
Urothelial	8 (3.40%)
Collecting duct	1 (0.43%)
Sarcomatic	2 (0.85%)
Chromophobe	20 (8.51%)
Clear cell	184 (78.30%)
Planoepithelial	4 (1.70%)
Oncocytoma	3 (1.28%)
Cystic	2 (0.85%)
Chromophobe + Clear cell	7 (2.98%)
Papillary	1 (0.43%)
Postoperative complications	
No	176 (74.89%)
Yes	59 (25.11%)
Postoperative complications according to Clavien–Dindo classification	
I	0 (0%)
II	2 (0.85%)
IIIa	0 (0%)
IIIb	2 (0.85%)
IVa	37 (15.75%)
IVb	11 (4.68%)
V	7 (2.98%)
30-day mortality	7 (2.98%)
Follow-up time (months)	22 (0–159) IQR 42.5
Follow-up mortality	119 (50.64%)

**Table 2:** The general patient characteristics according to thrombus level.

Variable	VTT 1 (*n* = 123)	VTT 2–4 (*n* = 112)	Total (*n* = 235)	*p*
Age	66 (30–86) IQR 13.5	67 (32–87) IQR 14	67 (30–87) IQR 14	0.34
Gender				0.07
Male	79 (70.53%)	73 (59.35%)	152 (64.68%)	
Female	33 (29.46%)	50 (46.65%)	83 (35.19%)	
BMI (kg/m^2^)	26.73 (18.59–40.9) IQR 6.33	25.31 (18.03–49.27) IQR 6.56	25.87 (18.03–49.27) IQR 6.28	0.03
Comorbidities (yes)	66 (58.93%)	61 (49.60%)	127 (54.04%)	0.15
NRS	3 (2–6) IQR 1	4 (2–6) IQR 2	4 (2–6) IQR 1	<0.001
CCI points	6 (2–11), IQR 3	7 (3–12) IQR 4	7 (2–12) IQR 4	0.05
ASA score	2 (1–3) IQR 0	2 (1–3) IQR 0.5	2 (1–3) IQR 0	0.41
Tumor side				0.001
Right	52 (46.43%)	83 (67.48%)	135 (57.45%)	
Left	60 (53.57%)	40 (32.52%)	100 (42.55%)	

Abbreviations: ASA—American Society of Anesthesiologists, BMI—Body mass index, CCI—Charlson Comorbidity Index, IQR—Interquartile range, NRS—Nutritional risk score.

**Table 3:** Laboratory test results according to thrombus level.

Variable	VTT 1 (*n* = 123)	VTT 2–4 (*n* = 112)	Total (*n* = 235)	*p*
	Preoperative level			
Hemoglobin (g/dL)	12.21 (7.5–17.4) SD 2.17	11.15 (5.1–17.3) SD 2.21	11.8 (5.1–17.4) IQR 3.18	<0.001
WBC (G/L)	7.88 (3.25–23.71) IQR 3.16	7.89 (2.5–64.21) IQR 3.88	7.88 (2.5–64.21) IQR 3.47	0.99
Lymphocytes (G/L)	1.89 (0.31–12.48) IQR 3.37	2.14 (0.55–59.31) IQR 3.96	1.95 (0.31–59.41) IQR 3.58	0.71
Neutrophiles (G/L)	3.61 (0.63–21.27) IQR 4.36	3.52 (0.74–78.9) IQR 4.29	3.57 (0.63–78.9) IQR 4.33	0.59
Monocytes (G/L)	0.65 (0.27–1.93) IQR 0.35	0.71 (0.11–7.6) 0.32	0.68 (0.11–7.6) IQR 0.39	0.23
PLT (G/L)	257.5 (88–763) IQR 108.5	257.5 (76–803) IQR 167.5	257.5 (76–803) IQR 141	0.77
MLR	0.30 (0.06–1.77) IQR 0.31	0.34 (0.01–5.13) IQR 0.42	0.32 (0.01–5.14) IQR 0.39	0.32
NLR	2.21 (0.09–18.33) IQR 3.53	2.3 (0.07–53.31) IQR 4.24	2.25 (0.07–53.31) IQR 4.18	0.87
PLR	116.43 (19.81–600) IQR 147.88	120.31 (2.72–658.20) IQR 174.03	117.12 (2.72–658.20) IQR 168.91)	0.66
Creatinine (µmol/L)	93.29 (48.67–898.42) IQR 36.53	97.82 (43.99–547.9) IQR 41.87	93.9 (43.99–89.42) IQR 38	0.15
Sodium (mmol/L)	139.1 (124.5–144.9) IQR 3.3	138 (105.5–145.6) IQR 5.12	138.8 (105.5–145.6) IQR 3.6	0.048
Potassium (mmol/L)	4.5 (2.99–5.8) IQR 0.5	4.5 (3.2–6) IQR 0.62	3.5 (2.99–6) IQR 0.6	0.73
	Postoperative level			
Hemoglobin (g/dL)	10.45 (6.8–15.2) IQR 2.83	9.6 (5.5–14.5) IQR 2.1	9.9 (5.5–15.2) IQR 2.8	<0.001
WBC (G/L)	11.11 (4.22–23.07) IQR 4.55	10.19 (2.26–142.2) IQR 4.7	10.61 (2.26–142.2) IQR 4.74	0.15
Lymphocytes (G/L)	1.68 (0.24–14.5) IQR 4.38	1.98 (0.18–44.98) IQR 6.83	1.73 (0.18–44.98) IQR 5.48)	0.18
Neutrophiles (G/L)	6.29 (0.5–25.82) IQR 7.48	5.13 (0.47–17.86) IQR 7.92	5.69 (0.47–25.82) IQR 7.81	0.65
Monocytes (G/L)	0.85 (0.3–2.28) IQR 0.45	0.82 (0.17–4.63) IQR 0.43	0.84 (0.17–4.63) IQR 0.45	0.55
PLT (G/L)	220 (21–530) IQR 99.75	216.5 (25.6–703) IQR 129.25	220 (21–703) IQR 110.5	0.94
MLR	0.54 (0.01–2.47) IQR 0.71	0.47 (0.02–3.7) IQR 0.69	0.52 (0.01–3.70) IQR 0.71	0.24
NLR	4.41 (0.01–92.44) IQR 7.91	3.09 (0.07–56.61) IQR 7.35	4.07 (0.01–92.45) IQR 7.68	0.35
PLR	117.73 (7.52–1271.79) IQR 182.3	85.37 (2.69–1738.89) IQR 197.07	102.37 (2.69–1738.89) IQR 187.05	0.27
Creatinine (µmol/L)	122.25 (57.55–738.05) IQR 45.12	119.415 (59–1065.88) IQR 55.34	115.65 (57.55–1065.88) IQR 46.39	0.44
Sodium (mmol/L)	139 (129.9–149.2) IQR 3.45	138.4 (129–169) IQR 4.08	138.7 (129–169) IQR 3.5	0.24
Potassium (mmol/L)	4.3 (2.94–6.2) IQR 0.79	4.3 (2.7–5.9) IQR 0.7	4.3 (2.7–6.2) IQR 0.8	0.80

Abbreviations: IQR—Interquartile range, MLR—Monocytes-Lymphocytes ratio, NLR—Neutrophils-Lymphocytes ratio, PLT—Platelets, PLR—Platelets-Lymphocytes ratio, SD—Standard deviation.

**Table 4:** Intraoperative and early postoperative characteristics according to thrombus level.

Variable	VTT 1 (*n* = 123)	VTT 2–4 (*n* = 112)	Total (*n* = 235)	*p*
Procedure time (min)	107.5 (40–230) IQR 50	125 (45–285) IQR 56.25	115 (40–285) IQR 55	0.06
Blood loss (mL)	264.11 (0–2500) SD 54.51	599.18 (0–4000) SD 85.32	442.25 (0–4000) SD 797.74	0.001
Red blood cells transfusion (units)	0.69 (0–4) SD 1.12	1.46 (0–10) SD 1.97	1.09 (0–10) IQR 1.67	<0.001
Surgery type				0.37
Nephrectomy	111 (99.11%)	119 (96.75%)	230 (97.87%)	
Laparotomy	1 (0.89%)	4 (3.25%)	5 (2.13%)	
Adrenalectomy	69 (61.61%)	87 (70.73%)	156 (66.38%)	0.14
Lymphadectomy	24 (21.43%)	31 (25.20%)	55 (23.40%)	0.49
Hospitalization time (days)	8 (3–32) IQR 3	9 (2–39) IQR 4	9 (2–39) IQR 4	<0.001
Postoperative hospitalization time (days)	6 (0–30) IQR 2	7 (0–27) IQR 3	7 (0–30) IQR 3	<0.001
Complications	15 (13.39%)	44 (35.77%)	59 (25.11%)	<0.001
Hemorrhagic shock	11 (9.82%)	32 (26.06%)	43 (18.29%)	0.001
Acute respiratory and circulatory insufficiency	2 (1.79%)	4 (3.25%)	6 (2.55%)	0.69
Preoperative pulmonary embolism	0 (0%)	3 (2.46%)	3 (1.28%)	0.24
Ischemic stroke	0 (0%)	4 (3.25%)	3 (1.28%)	1
Cardiac arrest	1 (0.89%)	2 (1.62%)	3 (1.28%)	0.62
Postoperative pulmonary embolism	0 (0%)	2 (1.62%)	2 (0.85%)	0.49
Renal insufficiency requiring dialysis	0 (0%)	2 (1.62%)	2 (0.85%)	0.24
Intraabdominal hematoma	0 (0%)	2 (1.62%)	2 (0.85%)	0.49
Sepsis	0 (0%)	2 (1.62%)	2 (0.85%)	0.29
Atonia of the digestive tract	0 (0%)	1 (0.81%)	1 (0.43%)	1
Hemorrhage from urinary tract	1 (0.89%)	0 (0%)	1 (0.43%)	0.48
Iatrogenic digestive tract perforation	0 (0%)	1 (0.81%)	1 (0.43%)	1
Cardiogenic shock	1 (0.89%)	0 (0%)	1 (0.43%)	0.29
Intraoperative pulmonary embolism	0 (0%)	1 (0.81%)	1 (0.43%)	0.49
30-day mortality	2 (1.79%)	5 (4.06%)	7 (2.98%)	0.45
Complications according to Clavien–Dindo classification				
Minor (I–II)	1 (0.89%)	3 (2.46%)	4 (1.70%)	<0.001
Major (III–V)	14 (12.50%)	41 (33.33%)	55 (23.40%)	
I	0 (0%)	0 (0%)	0 (0%)	0.004
II	1 (0.89%)	1 (0.81%)	2 (0.85%)	
IIIa	0 (0%)	0 (0%)	0 (0%)	
IIIb	0 (0%)	2 (1.62%)	2 (0.85%)	
IVa	10 (8.93%)	27 (21.95%)	37 (15.74%)	
IVb	2 (1.79%)	9 (7.32%)	11 (4.68%)	
V	2 (1.79%)	5 (4.07%)	7 (2.98%)	

Abbreviations: IQR—Interquartile range, SD—Standard deviation.

**Table 5:** Univariate logistic regression analysis of predictive factors for postoperative complications.

Variable	Odd Ratio	95% Confidence Interval	*p*
Age	0.99	0.96–1.02	0.75
Gender			
Female	1	1	0.19
Male	1.53	0.81–2.90	
BMI	0.97	0.91–1.04	0.52
Comorbidities	1.38	0.76–2.51	0.28
NRS	4.15	2.73–6.30	<0.001
CCI points	1.07	0.94–1.22	0.30
Level of tumor vein invasion			
Level 1	1	1	<0.001
Level 2–4	3.73	1.94–7.19	
Procedure time	1.02	1.004–1.02	0.001
Blood loss	1.002	1.001–1.002	<0.001
Hemoglobin	0.79	0.69–0.91	0.001
White Blood Cells	1.08	1.008–1.15	0.04
Lymphocytes	0.82	0.70–0.95	0.008
Neutrophiles	1.19	1.09–1.32	<0.001
Monocytes	2.51	0.99–6.36	0.07
MLR	4.86	1.94–12.15	0.001
NLR	1.18	1.09–1.29	<0.001
PLR	1.002	1.001–1.005	0.003
Platelets	1.001	0.99–1	0.18
Creatinine	1	0.99–1	0.28
Sodium	0.99	0.92–1.07	0.91
Potassium	0.86	0.47–1.58	0.73

Abbreviations: BMI—Body mass index, CCI—Charlson Comorbidity Index, MLR—Monocytes-Lymphocytes ratio, NLR—Neutrophils-Lymphocytes ratio, NRS—Nutritional risk score, PLR—Platelets-Lymphocytes ratio.

**Table 6:** Tumor characteristics.

Variable	Level 1	Level 2–4	*p*
Tumor size (cm)	7.5 (0.2–23) IQR	8.75 (0.8–24)	0.001
TNM			
T			
T1a	4	0	0.05
T1b	10	1	0.003
T2a	9	4	0.15
T3a	69	80	0.59
T3b	3	8	0.22
T3c	0	1	1
T4	4	18	0.003
N			
N0	99	105	0.56
N1	11	12	1
N2	2	6	0.29
M			0.52
M0	106	119	
M1	6	4	
Histopathological type			
Fuscocellular + Clear cell	1 (0.89%)	0 (0%)	0.48
Fuscocellular	1 (0.89%)	1 (0.81%)	1
Urothelial	4 (3.57%)	4 (3.25%)	1
Collecting duct	1 (0.89%)	0 (0%)	0.48
Sarcomatic	1 (0.89%)	1 (0.81%)	1
Chromophobe	12 (10.71%)	8 (6.50%)	0.25
Clear cell	85 (75.89%)	99 (80.50%)	0.39
Planoepithelial	2 (1.79%)	2 (1.62%)	1
Oncocytoma	2 (1.79%)	1 (0.81%)	0.61
Cystic	1 (0.89%)	1 (0.81%)	1
Chromophobe + Clear cell	2 (1.79%)	5 (4.06%)	0.44
Papillary	0 (0%)	1 (0.81%)	1

Abbreviations: IQR—Interquartile range.

**Table 7:** Follow-up data.

Variable	Level 1	Level 2–4	*p*
Follow-up time (months)	30 (0–144) IQR 40.25	16 (0–159) IQR 36	0.001
Mortality	39 (34.82%)	80 (65.04%)	<0.001
1-year overall survival	83.61% SE 3.53%	59.65% SE 4.44%	<0.001
5-year overall survival	60.24% SE 5.29%	27.41% SE 4.73%	<0.001

Abbreviations: IQR—Interquartile range, SE—Standard error.

**Table 8:** Univariate Cox proportional hazard regression analysis (first model).

Variable	Hazard Ratio	95% Confidence Interval	*p*
Age	1.006	0.99–1.02	0.49
Gender			
Female	1		0.85
Male	0.96	0.66–1.40	
BMI	0.94	0.90–0.99	0.01
NRS 2002	1.38	1.15–1.65	<0.001
Comorbidities	1.005	0.70–1.44	0.98
CCI score	1.22	1.11–1.33	<0.001
Level of tumor vein invasion			
Level 1	1		<0.001
Level 2–4	2.40	1.64–3.53	
Procedure time	1.003	0.99–1.008	0.16
Blood loss	1.0004	1.000–1.0001	<0.001
Preoperative hemoglobin	0.77	0.71–0.83	<0.001
Preoperative white blood cells	1.01	0.99–1.04	0.26
Preoperative lymphocytes	1.003	0.97–1.04	0.88
Preoperative neutrophiles	1.004	0.98–1.03	0.69
Preoperative monocytes	1.20	1.007–1.43	0.04
Preoperative platelets	1.003	1.002–1.004	<0.001
Preoperative MLR	1.26	0.98–1.63	0.07
Preoperative NLR	1.01	0.98–1.04	0.43
Preoperative PLR	1.002	1.001–1.003	0.001
Preoperative creatinine	0.99	0.99–1.001	0.57
Preoperative sodium	0.93	0.90–0.97	<0.001
Preoperative potassium	1.88	1.33–2.63	<0.001
Postoperative hemoglobin	0.75	0.68–0.83	<0.001
Postoperative white blood cells	1.02	1.001–1.04	0.04
Postoperative lymphocytes	1.01	0.98–1.05	0.38
Postoperative neutrophiles	0.99	0.95–1.04	0.98
Postoperative monocytes	1.51	1.008–2.25	0.045
Postoperative platelets	1.003	1.001–1.005	<0.001
Postoperative MLR	1.08	0.73–1.58	0.71
Postoperative NLR	0.99	0.98–1.02	0.73
Postoperative PLR	1	0.99–1.001	0.25
Postoperative creatinine	0.99	0.99–1.001	0.57
Postoperative sodium	0.91	0.86–0.96	<0.001
Postoperative potassium	1.58	1.16–2.16	<0.001
Postoperative complications	2.26	1.54–3.32	<0.001
Tumor size	1.09	1.04–1.13	<0.001
T			
1a	0.34	0.04–2.49	0.29
1b	0.32	0.08–1.29	0.11
2	0.76	0.33–1.75	0.52
3a	1		
3b	0.86	0.37–1.99	0.74
4	1.82	1.10–2.99	0.02
N			
0	1		0.002
1–2	1.58	1.17–2.14	
M			
0	1		
1	2.58	1.25–5.31	0.01

Abbreviations: BMI—Body mass index, CCI—Charlson Comorbidity Index, MLR—Monocytes-Lymphocytes ratio, NLR—Neutrophils-Lymphocytes ratio, NRS—Nutritional risk score, PLR—Platelets-Lymphocytes ratio.

**Table 9:** Univariate Cox proportional hazard regression analysis (second model).

Variable	Hazard Ratio	95% Confidence Interval	*p*
Age	1.006	0.99–1.02	0.49
Gender			
Female	1		0.85
Male	0.96	0.66–1.40	
BMI	0.94	0.90–0.99	0.01
NRS 2002	1.38	1.15–1.65	<0.001
Comorbidities	1.005	0.70–1.44	0.98
CCI score	1.22	1.11–1.33	<0.001
Level of tumor vein invasion			
Level 1	1		<0.001
Level 2–4	2.40	1.64–3.53	
Procedure time	1.003	0.99–1.008	0.16
Blood loss	1.0004	1.000–1.0001	<0.001
Preoperative hemoglobin	0.77	0.71–0.83	<0.001
Preoperative white blood cells	1.01	0.99–1.04	0.26
Preoperative lymphocytes	1.003	0.97–1.04	0.88
Preoperative neutrophiles	1.004	0.98–1.03	0.69
Preoperative monocytes	1.20	1.007–1.43	0.04
Preoperative platelets	1.003	1.002–1.004	<0.001
Preoperative MLR	1.26	0.98–1.63	0.07
Preoperative NLR	1.01	0.98–1.04	0.43
Preoperative PLR	1.002	1.001–1.003	0.001
Preoperative creatinine	0.99	0.99–1.001	0.57
Preoperative sodium	0.93	0.90–0.97	<0.001
Preoperative potassium	1.88	1.33–2.63	<0.001
Tumor size	1.09	1.04–1.13	<0.001
T			
1a	0.34	0.04–2.49	0.29
1b	0.32	0.08–1.29	0.11
2	0.76	0.33–1.75	0.52
3a	1		
3b	0.86	0.37–1.99	0.74
4	1.82	1.10–2.99	0.02
N			
0	1		0.002
1–2	1.58	1.17–2.14	
M			
0	1		
1	2.58	1.25–5.31	0.01

Abbreviations: BMI—Body mass index, CCI—Charlson Comorbidity Index, MLR—Monocytes-Lymphocytes ratio, NLR—Neutrophils-Lymphocytes ratio, NRS—Nutritional risk score, PLR—Platelets-Lymphocytes ratio.

## Data Availability

The raw data supporting the conclusions of this article will be made available by the authors on request.
